# Editorial: Utilizing real world data and real world evidence in veterinary medicine: current practices and future potentials

**DOI:** 10.3389/fvets.2025.1745155

**Published:** 2026-05-21

**Authors:** Andrea Wright, Kennedy Mwacalimba, Taran Rai

**Affiliations:** 1 Global Medical Affairs, Clinical Studies and Outcomes Research, Zoetis Inc, Parsippany, NJ, United States; 2 Surrey DataHub, vHive, School of Veterinary Medicine, University of Surrey, Guildford, United Kingdom; 3 Centre for Vision, Speech and Signal Processing, University of Surrey, Guildford, United Kingdom

**Keywords:** veterinary medicine, regulatory, real real-world data (RWD), Real real-world evidence (RWE), artificial intelligence

## 1 Introduction

Outside the silo of controlled trials, the daily realities of veterinary practice within a regulated environment generate a wealth of real-world evidence (RWE)—capturing how interventions work across diverse patients and contexts, and offering insights essential for informed care, responsible surveillance, and public health. By drawing on real-world data from sources such as electronic health records, laboratory results, owner surveys, social media posts, registries, and price repositories, RWE provides the external validity needed to complement clinical trials, inform practice, strengthen surveillance, and guide policy across companion animals, livestock, and One Health. The studies in this Research Topic showcase how such diverse data can be transformed into decision-relevant insights, spanning clinical practice, data infrastructure and artificial intelligence, economics and policy, and cross-border surveillance. Together, they illustrate the growing potential of RWD and RWE in advancing veterinary medicine within a regulatory context, a perspective further explored by Bruno.

Advances in artificial intelligence (AI), including large language models (LLMs), are rapidly expanding how veterinary real-world data are interpreted and used. These tools enable efficient analysis of unstructured information such as electronic health records and owner-reported data, turning everyday clinical observations into actionable evidence.

The Research Topic of articles has been grouped into thematic insights.

### 1.1 Practice-based evidence

Evidence from daily practice offers direct insights into patient care and owner–clinician interactions. A cross-sectional, US practice-based CKD study combining veterinarian- and owner-reported data showed most dogs were diagnosed at earlier IRIS stages and underscored frequent dietary recommendations and symptom management in routine care. Agreement analyses between owners and veterinarians revealed variable concordance across clinical signs and highlighted misalignments regarding which signs owners found most problematic. This information is directly useful for consultation design and client education (Wright et al.). The perceptions of swine veterinarians in Europe were collected pertaining to evidence-based veterinary medicine. This study identified the need for more education and practical frameworks for application in practice (Teixeira Costa et al.).

### 1.2 Building data infrastructures for surveillance

Linked national databases provide powerful tools for surveillance and antimicrobial stewardship. A Swiss study combining production, movement, and antibiotic use data successfully classified farms into production types, enabling targeted surveillance and benchmarking of antimicrobial consumption (Schnidrig et al.). Similarly, research on Kenya's surveillance systems demonstrated the value of secondary data in detecting zoonotic threats (Kahariri et al.), while highlighting the need for standardized tools such as the Surveillance and Information Sharing Operational Tool (SIS-OT).

### 1.3 Electronic health records as real-world data

Electronic health records (EHRs) are emerging as foundational for veterinary pharmacoepidemiology. Using GPT-4o to extract six feline clinical signs from referral hospital EHRs achieved high sensitivity, specificity, and inter-run reproducibility. Most discrepancies occurred in records where human reviewers also disagreed, indicating ambiguity rather than systematic model errors. The work also quantified operational factors (temperature settings, cost/time) relevant for deployment (Wulcan et al.). Using large language models (LLMs) complements broader efforts to develop EHRs as a primary data source for veterinary pharmacoepidemiology in the UK (Davies et al.). This proof-of-concept study demonstrated that first opinion EHRs from large unstructured data sets can be used to identify adverse events. Petrou et al. used first opinion EHRs for identification of medication errors, representing new research into medication safety in veterinary medicine. Pharmacoepidemiology is well established in human health, ever evolving to control bias, using artificial intelligence to advance learning from unstructured data. O'Rourke et al. paired EHRs with activity monitors and environmental factors to investigate activity levels in North American dogs.

### 1.4 Economics, and organization of care

Economic analyses reveal important patterns in veterinary markets. A cross-country price study of 771 clinics in Sweden and Norway showed significant differences by country, clinic type, and corporate affiliation, with chains pricing higher than independent practices (Egenvall et al.). In parallel, a US SWOT analysis argues that with focused investment in staff utilization, technology adoption, mission clarity, and improved access to capital, independent practices can remain competitive and resilient in a changing market (Traub-Werner et al.). Together, these studies demonstrate how RWD can inform debates on price transparency, access to care, and organizational resilience.

### 1.5 One health and transboundary risk

RWD also plays a critical role in One Health surveillance. This role is evident in a structured risk assessment of African Swine Fever (ASF) across 40 territories in the Americas, which highlighted both clear and uncertain pathways of incursion. While some regions faced probable risk through informal and legal imports, many others remained difficult to classify due to gaps in available data. These findings highlight the need to strengthen border biosecurity, enhance waste stream management in sectors such as cruise and aviation, and expand regional data sharing to convert uncertainty into targeted prevention (Arcega Castillo et al.). Beyond disease surveillance, capacity-building in veterinary medical product regulation, particularly around antimicrobial resistance, remains a shared priority across human and animal health domains (Pyatt et al.).

### 1.6 Methodological advances

A two-decade laboratory dataset, analyzed with Bayesian methods, provided credible interval estimates of breed-stratified prevalence of hyposegmentation of granulocytes/Pelger-Huët anomaly and signaled newly affected breeds. This demonstrates how probabilistic approaches can deliver robust estimates for rare outcomes in operational settings (Carli et al.).

## 2 Methods, data lessons and sources

Pet owner survey data on canine chronic kidney disease, developed by veterinary experts, was complemented by new insights on pain derived from social media listening (Tarrant et al.). In a different study using social media data, the challenges of administering oral medications to dogs were identified globally (Tarrant et al.) Different methods can harmonize perspectives to improve care by matched clinician–owner reporting with agreement statistics surfacing communication gaps and guiding symptom-monitoring priorities in chronic conditions like CKD (Wright et al.). Models with context, such as mixed-effects modeling with log-transformed, clinic-level price data and explicit random effects, can fairly account for service configuration and affiliation when benchmarking markets (Egenvall et al.). Engineered features for surveillance created linked registries enriched with production and movement features support high-accuracy machine learning that translates directly into targeted surveillance and stewardship actions (Schnidrig et al.). Operationalizing EHR text by validating LLM extraction against multi-reviewer human references and characterizing errors by temporal/qualitative ambiguity helps right-size expectations and standardize workflows for retrospective studies (Wulcan et al.) These advances illustrate how AI methods are being applied to transform unstructured veterinary data into reliable, scalable evidence for research and surveillance. Bayesian approaches are suited to sparse-event RWD, converting long-horizon lab archives into actionable prevalence estimates with explicit credible intervals (Carli et al.). Governance matters: De-identification, secure API configurations, and local approvals remain essential for scale and cross-institutional generalizability when using EHR-derived RWD (Wulcan et al.).

## 3 Implications

Clinicians and practice leaders should embed owner-facing education and monitoring plans for high-impact signs (e.g., CKD polyuria/polydipsia, appetite changes) and integrate routine dietary recommendations guided by observed adherence and burden in real-world settings (Wright et al.). Validated EHR extraction pipelines should be used to accelerate audits and retrospective analyses, paying attention to operational parameters (e.g., temperature settings) and local validation before scale-up (Wulcan et al.).

For industry and markets there is a need to support price transparency and market benchmarking by pairing price data with clinic characteristics and affiliation metadata; this informs fair comparisons and purchasing decisions across settings (Egenvall et al.). The ability to strengthen independent practices through targeted technology adoption, better staff utilization, and clear mission communication to sustain competition, access, and innovation (Traub-Werner et al.)

For public and animal health authorities direct ASF prevention resources toward high-risk pathways (informal imports, traveler-carried goods, poorly managed waste streams) and collaborate regionally to close information gaps that drive “Unknown” classifications (Arcega Castillo et al.). Additionally expand linked-data programs to classify production systems and target surveillance and antimicrobial stewardship with greater precision (Schnidrig et al.).

## 4 Future priorities

In the near-term, we should standardize reporting templates and minimum datasets for practice-based RWD studies (e.g., signs, staging, outcomes, owner-reported measures) to improve comparability across clinics and studies (Wright et al.). Publish clinic metadata with price observations (e.g., hours, on-call, hospital status) to sharpen fair-market comparisons and transparency initiatives(Egenvall et al.).

In the medium-term we need to scale validated EHR extraction across institutions and species; share prompt/instruction sets and parameter configurations to enhance reproducibility and cost-efficiency (Wulcan et al.). Extend linked-data ML classification beyond cattle and incorporate antimicrobial and movement outcomes to operationalize risk-based surveillance programs (Schnidrig et al.).

Longer-term the veterinary and animal health stakeholders should build federated, well-governed RWD ecosystems across EHRs, labs, registries, sensors, and claims to power pharmacoepidemiology and quality measurement (Davies et al.) and normalize Bayesian and causal-inference toolkits for sparse-event, policy-relevant veterinary questions, including safety and effectiveness estimation under uncertainty (Carli et al.). Institutionalize regional One Health data exchanges that continuously update transboundary risk classification and intervention targeting (Arcega Castillo et al.).

## 5 Limitations

Generalizability is conditioned by context: EHR-based findings derive from a single tertiary referral hospital; price analyses focus on Sweden and Norway; ASF risk classification relies on multi-source evidence with variable depth across territories. These underscore the need for multi-institutional validation, broader geographic sampling, and structured data-sharing to translate insights globally (Wulcan et al.; Egenvall et al.; Arcega Castillo et al.).

## 6 Conclusion

This Research Topic demonstrates how veterinary real-world data—collected beyond the silo of controlled trials from clinics, registries, laboratories, web platforms, and EHR free text—can be transformed into real-world evidence that meaningfully informs clinical practice, market transparency, surveillance, and policy. To fully realize this potential, advancing the agenda will require shared reporting standards, reproducible analytical pipelines, and responsible data governance across sectors. The contributions here provide concrete methods and early exemplars to scale what works next, bridging the gap between controlled trial evidence and the complex realities of veterinary care.

